# Validation of self-reported anthropometrics in the Adventist Health Study 2

**DOI:** 10.1186/1471-2458-11-213

**Published:** 2011-04-05

**Authors:** Maira Bes-Rastrollo, Joan Sabaté, Karen Jaceldo-Siegl, Gary E Fraser

**Affiliations:** 1 Dept. of Preventive Medicine and Public Health, School of Medicine, University of Navarra, Pamplona, Spain; 2 Dept. of Nutrition, School of Public Health, Loma Linda University, Loma Linda, CA, USA; 3 Dept. of Epidemiology and Statistics. School of Public Health, Loma Linda University, Loma Linda, CA, USA

## Abstract

**Background:**

Relying on self-reported anthropometric data is often the only feasible way of studying large populations. In this context, there are no studies assessing the validity of anthropometrics in a mostly vegetarian population. The objective of this study was to evaluate the validity of self-reported anthropometrics in the Adventist Health Study 2 (AHS-2).

**Methods:**

We selected a representative sample of 911 participants of AHS-2, a cohort of over 96,000 adult Adventists in the USA and Canada. Then we compared their measured weight and height with those self-reported at baseline. We calculated the validity of the anthropometrics as continuous variables, and as categorical variables for the definition of obesity.

**Results:**

On average, participants underestimated their weight by 0.20 kg, and overestimated their height by 1.57 cm resulting in underestimation of body mass index (BMI) by 0.61 kg/m^2^. The agreement between self-reported and measured BMI (as a continuous variable), as estimated by intraclass correlation coefficient, was 0.97. The sensitivity of self-reported BMI to detect obesity was 0.81, the specificity 0.97, the predictive positive value 0.93, the predictive negative value 0.92, and the Kappa index 0.81. The percentage of absolute agreement for each category of BMI (normoweight, overweight, and obese) was 83.4%. After multivariate analyses, predictors of differences between self-reported and measured BMI were obesity, soy consumption and the type of dietary pattern.

**Conclusions:**

Self-reported anthropometric data showed high validity in a representative subsample of the AHS-2 being valid enough to be used in epidemiological studies, although it can lead to some underestimation of obesity.

## Background

Obesity is one of the main public health problems of this century. The prevalence of overweight and obesity has reached epidemic proportions around the world, not only in developed countries but also in developing countries. Thus, it has been recognised as a pandemic by the World Health Organization [1].

Obesity is an important risk factor for several chronic diseases such as cardiovascular disease, hypertension, type 2 diabetes, arthritis, and some cancers [2]. In addition, the causes of obesity are multiple and complex. For all these reasons it is important to assess obesity as a risk factor and to study the potential causes of weight gain in large populations.

Body mass index (BMI) has been used as a measure of obesity, with obesity defined as a BMI ≥30 kg/m^2 ^(calculated as weight in kg divided by height in meters squared) [1]. Self-reported height and weight provide a cost-effective assessment of these variables and thus have been used in numerous epidemiological studies as the only feasible way to obtain information. However, relying on self-reported data of participants has inherent problems. Therefore, it is of the utmost importance to assess the validity of that information.

The validity of "self-reported" BMI, calculated from self-reported height and weight has been assessed in several populations [3-6]. Results vary depending on the characteristics of the population. In this context, there are no studies assessing the validity of these self-reported measures in a population mostly vegetarian. Therefore, our objective was to evaluate the validity of self-reported anthropometrics in a representative subsample of the Adventist Health Study 2 (AHS-2) cohort.

## Methods

### The Adventist Health Study 2 (AHS-2)

The AHS-2 is a prospective cohort study of over 96,000 adult Adventists in the USA and Canada. The main objective of the study is to assess the association between lifestyle choices, particularly diet, and the risk of cancer and other health outcomes. Its methods have been described elsewhere [7]. Briefly, the sample includes Seventh-day Adventist church members living in the USA and Canada who were 30 years and older, and who were sufficiently fluent in English to complete the lengthy lifestyle questionnaire. Enrolment started in February 2002 and finished December 2007. All participants completed a self-reported lifestyle questionnaire including their weight and height. Most participants were either Caucasian or Black/African-American. The AHS-2 cohort is relatively unique for its wide range of dietary patterns compared with the general Western population. Also interesting is that about half of participants practice some kind of vegetarian dietary pattern [8].

### Validation study

Subjects included in this report are part of a calibration study for the AHS-2 conducted at Loma Linda University. Participants were randomly selected from the AHS-2 using a two-stage random selection method: first, churches were randomly selected from around North America, being weighted for church size; then subjects were randomly chosen from each of these churches. Participants attended a scheduled clinic appointment at their local church. Weight was measured with a calibrated scale without shoes and heavy outer garments, to the nearest 0.1 kg and height was measured with a stadiometer without shoes to the nearest 1/4 inch. Both measurements were taken by a trained research assistant. Vegetarian status, and intake of soy and nuts were obtained from a previously validated food frequency questionnaire [9]. Participants with a diagnosis of active cancer, Alzheimer's disease, or pregnant women were excluded from the calibration study. Unqualified subjects and those who refused to take part in the calibration study (about one third) were replaced with individuals randomly chosen from the same church and matched by race, age (within 5 y of the original subject age), and sex. Because of the special focus on black Adventist as a minority in the AHS-2, the calibration was designed to include approximately equal numbers of blacks and whites. The study was approved by the institutional review board of Loma Linda University, and all subjects signed a written informed consent.

### Statistical analyses

We calculated the crude differences between self-reported and measured anthropometrics (weight, height, and BMI) and adjusted them for time-lag between self-reported and measured anthropometric data, and for race, age, and sex by analyses of covariance. We report the differences between self-reported and measured variables according to population characteristics and compared them through Student T tests, or analyses of variance, depending on the form of the variables.

We studied the agreement between self-reported and measured anthropometric variables using a random-effect model intraclass correlation coefficient and the survival agreement plot for BMI proposed by Luiz et al. [10] differentiating between negative and positive differences [11].

Non-parametric Spearman correlation coefficients were calculated between self-reported and measured anthropometric variables. Sensitivity, specificity, predictive positive and negative values and Kappa index were calculated for the validation of obesity (BMI≥30 kg/m^2^).

Participants were categorised into quintiles of weight according to their self-reported and measured weight information. The percentages of misclassification in different adjacent quintiles or, when the misclassification was more than two quintiles, were estimated. Similarly, we classified participants according to each category of BMI (normal weight, overweight and obese) and calculated the absolute agreement and the overall kappa index.

We also present the BMI relative error (self-reported-measured/measured*100) graphed against the average BMI according to Bland and Altman [12].

Finally, to evaluate predictors of differences between self-reported and measured weight and BMI, multivariate linear regression analyses were performed taking weight and BMI differences [self-reported-measured] as the dependent variable.

## Results

From 1011 participants in the calibration study, we excluded one hundred participants with missing values, thus finally including 911 participants.

Those 911 participants were a good representation of the overall AHS-2cohort, except that the validation sample had a slightly higher percentage of soy consumers (Table 1). The difference in race is by design as the calibration study over-sampled black subjects.

**Table 1:** Characteristics of participants in the validation sample and in the Adventist Health Study-2 (AHS-2).

	AHS-2 (n = 95,681)	Validation Sample (n = 911)	p value	Adjusted p value for race^1^
Women (%)	65.0	66.7	0.28	0.98
Age (years)(SD)	58.6 (14.5)	58.3 (13.4)	0.58	0.02
Self-reported weight (kg) (SD)	76.7 (19.0)	77.8 (18.2)	0.09	0.98
Self-reported height (m) (SD)	168.2 (0.1)	167.9 (0.1)	0.46	0.54
Body Mass Index (BMI)			0.12	0.93
Self-reported Overweight (%) (BMI	34.4	34.5		
≥25 kg/m^2 ^and BMI<30 kg/m^2^)				
Self-reported Obesity (%)	24.7	27.3		
(BMI ≥30 kg/m^2^)				
Ever smokers (%)	20.0	17.9	0.11	0.09
Marital status				
Married/Remarried (%)	70.9	71.6	0.66	0.002
Education			0.41	0.46
≤High School	22.5	21.4		
≤2-year college	39.5	41.7		
≤Doctoral degree	38.0	36.9		
Income per capita^2^			0.87	0.31
Low (%)	26.4	25.9		
Medium (%)	46.1	47.1		
High (%)	27.5	27.0		
Race			<0.001	NA
White (%)	65.2	46.9		
Black (%)	26.6	46.4		
Hispanic (%)	3.9	3.7		
Asian (%)	3.1	1.9		
Other (%)	1.2	1.1		
Physical activity^3^			0.74	0.25
Low (%)	17.6	17.5		
Medium (%)	30.8	31.9		
High (%)	51.7	50.6		
Soy consumers^4 ^(%)	72.9	76.7	0.02	0.004
Nuts consumption			0.28	0.43
≤1/week (%)	30.0	30.4		
2 to 4/week (%)	29.0	31.0		
≥5/week (%)	41.0	38.6		
Diet^5^			0.25	0.40
Non-vegetarian (%)	41.4	43.9		
Semi-vegetarian (%)	20.4	20.3		
Vegetarian (%)	38.2	35.8		

SD: Standard Deviation; NA: Not applicable.

Continuous variables are expressed as means and (standard deviation) and compare using a T-Student test. Categorical variables are expressed as percentages and compare using Chi-square test.

1) p value was obtained through multivariate linear regression for continuous variables and logistic regression for categorical variables.

2) Low: <10,000$, medium: ≥10,000 - <30,000$, High: ≥30,000$.

3) Low: <20 minutes of non-vigorous activity; medium: ≥20 minutes of non-vigorous activity; high: ≥20 minutes of vigorous activity.

4) Soy consumers: ≥1 g of soy protein consumption per day.

5) Semi-vegetarian: low meat consumption and pesco; Vegetarian: Vegan and Lactovegetarian.

On average, participants self-reported lower weight (-0.31 kg, 95% CI: -1.20 to +0.58), greater height (+1.66 cm, 95% CI: +0.99 to +2.33) and thus lower BMI (-0.64 kg/m^2^, 95% CI: -1.02 to -0.25) as compared to measured values, after adjusting for time-lag, measured anthropometrics, age and sex (Table 2).

**Table 2:** Comparison between self-reported and measured values for weight, height and body mass index (BMI) in the validation sample (n = 911) of the Adventist Health Study-2.

	Self-reported	Measured	Difference*	Adjusted^1 ^difference	Adjusted^2 ^difference
Weight (kg)	77.8	78.0	-0.20	-0.20	-0.31
	(76.6 - 78.9)	(76.7 - 79.2)	(-0.54 to +0.14)	(-0.54 to +0.14)	(-1.20 to +0.58)
Height (cm)	168	166	+1.57	+1.57	+1.66
	(167-169)	(166-167)	(+1.31 to +0.02)	(+1.31 to +1.82)	(+0.99 to +2.33)
BMI (kg/m^2^)	27.58	28.2	-0.61	-0.61	-0.64
	(27.18-27.97)	(27.75-28.61)	(-0.75 to -0.46)	(-0.75 to -0.61)	(-1.02 to -0.25)

BMI: Body Mass Index.

*Difference: self-reported - measured.

1) Adjusted for time-lag between self-reported and measured anthropometric variables through ANCOVA.

2) Adjusted for time-lag between self-reported and measured anthropometric variables, race, age, and sex through ANCOVA.

When we compared self-reported and measured anthropometrics according to population characteristics we found differences according to age: younger participants reported less weight than measured, however older participants reported more weight than objectively measured. Older participants reported more height than was measured. Those participants who reported less BMI were particularly between 45-55 years old. Obese participants also reported a more inaccurate BMI than non-obese participants reporting -0.9 kg/m^2 ^[95% CI: -1.3 to -0.6] less than measured. Similarly, those participants who were non-soy consumers (<1 g of soy protein per day) had more measurement error than soy-consumers (Table 3). There were no differences between sex, race, nut consumption and dietary pattern.

**Table 3:** Anthropometric differences according to population characteristics in the AHS-2 validation sample (n = 911).

	WEIGHT (kg) (95% CI)				HEIGHT (cm) (95% CI)				BODY MASS INDEX (kg/m2) (95% CI)			
	Self- reported	Measured	Difference^1^	p value	Self- reported	Measured	Difference^1^	p value	Self-reported	Measured	Difference^1^	p value
**Sex**				0.18				0.13				0.36
Women	74.5	74.9	-0.4		163	162	+1.4		27.9	28.6	-0.7	
(n = 608)	(73.1-76.0)	(73.4-76.4)	(-0.8 to +0.1)		(163-164)	(161-163)	(+1.1 to +1.8)		(27.4-28.5)	(28.0-29.2)	(-0.8 to -0.4)	
Men	84.2	84.1	+0.1		177	175	+1.8		26.9	27.4	-0.5	
(n = 303)	(82.4-86.1)	(82.1-86.1)	(-0.4 to +0.7)		(176-178)	(174-176)	(+1.5 to +2.2)		(26.3-27.4)	(26.8-28.0)	(-0.7 to -0.3)	

**Age (years)**				<0.001				<0.001				0.030
<45	78.1	79.1	-0.9		168	168	+0.6		27.6	28.2	-0.6	
(n:161)	(75.2-81.1)	(76.0-82.1)	(-1.8 to -0.04)		(167-170)	(166-169)	(+0.4 to +1.1)		(26.6-28.6)	(27.1-29.3)	(-1.0 to -0.3)	
45-<55	80.0	81.8	-1.9		169	168	+1.0		28.0	29.0	-1.0	
(n: 228)	(77.4-82.5)	(79.1-84.6)	(-2.6 to -1.1)		(168-171)	(167-169)	(+0.3 to +1.8)		(27.1-28.9)	(28.0-29.9)	(-1.3 to -0.6)	
55-<65	80.4	80.0	+0.4		169	167	+1.4		28.3	28.7	-0.4	
(n: 234)	(78.1-82.8)	(77.6-82.5)	(-0.2 to +1.0)		(167-170)	(166-168)	(+1.0 to +1.8)		(27.5-29.1)	(27.7-29.6)	(-0.6 to -0.1)	
65- <75	74.8	74.0	+0.8		166	164	+2.0		27.0	27.4	-0.4	
(n: 170)	(72.5-77.1)	(71.6-76.3)	(+0.2 to +1.4)		(165-168)	(163-166)	(+1.4 to +2.5)		(26.2-27.8)	(26.5-28.3)	(-0.7 to -0.1)	
≥75	72.0	70.6	+1.4		166	162	+3.4		26.1	26.8	-0.6	
(n: 118)	(69.1-74.9)	(67.7-73.5)	(+0.7 to +2.1)		(164-166)	(161-164)	(+2.8 to +4.0)		(25.2-27.0)	(25.8-27.7)	(-1.0 to -0.3)	

**Obesity**				0.27				0.91				0.03
Non-obese	70.4	70.5	-0.1		169	167	+1.6		24.7	25.1	-0.4	
(n: 662)	(69.5-73.3)	(69.5-71.5)	(-0.4 to +0.3)		(168-169)	(166-168)	(+1.3 to +1.9)		(24.4-24.9)	(24.9-25.4)	(-0.6 to -0.3)	
Obese	97.3	97.9	-0.6		166	1.64	+1.5		35.3	36.2	-0.9	
(n: 249)	(95.1-99.5)	(95.6-100.2)	(-1.4 to +0.3)		(165-167)	(163-166)	(+1.1 to +2.0)		(34.6-36.0)	(35.5-37.0)	(-1.3 to -0.6)	

**Race^2^**				0.11				0.92				0.07
White	75.0	74.8	+0.2		168	167	+1.5		26.4	26.9	-0.4	
(n = 427)	(73.4-76.7)	(73.1-76.6)	(-0.2 to +0.7)		(167-169)	(166-168)	(+1.3 to +1.8)		(25.9 -27.0)	(26.3-27.5)	(-0.6 to -0.3)	
Black	81.3	81.9	-0.6		168	166	+1.6		28.9	29.6	-0.7	
(n = 423)	(79.6-83.1)	(80.1-83.7)	(-1.1 to 0.0)		(167-169)	(165-167)	(+1.1 to +2.1)		(28.3-29.5)	(29.0 -30.3)	(-1.0 to -0.5)	
Hispanic	75.4	76.5	-1.1		166	164	+1.6		27.3	28.2	-0.9	
(n = 34)	(68.5-82.4)	(69.1-83.9)	(-2.8 to +0.59)		(162-170)	(161-168)	(+0.8 to +2.3)		(25.1-29.5)	(25.8-30.6)	(-1.6 to -0.3)	
Asian	61.3	60.1	+1.1		160	159	+1.0		23.9	23.9	+0.1	
(n = 17)	(55.6-67.0)	(53.5-66.8)	(-0.4 to +2.7)		(155-164)	(154-164)	(-0.1 to +2.0)		(22.2-25.8)	(21.5-26.2)	(-0.7 to +0.9)	

**Soy**												
**Consumer^3^**				0.001				0.72				0.001
No	79.6	80.9	-1.3		167	165	+1.4		28.7	29.7	-1.0	
(n = 168)	(76.8 -82.4)	(78.0 - 83.9)	(-2.2 to -0.4)		(165-168)	(164-167)	(+1.0 to +1.9)		(27.7-29.7)	(28.6-30.8)	(-1.4 to -0.7)	
Yes	76.8	76.5	+0.3		168	167	+1.4		27.1	27.4	-0.4	
(n = 552)	(75.3 -78.3)	(74.9 - 78.1)	(-0.2 to +0.7)		(168-169)	(166-168)	(+1.1 to +1.7)		(26.6-27.5)	(26.9-28.0)	(-0.6 to -0.2)	

**Nut Intake**				0.74				0.92				0.58
Low	79.7	80.0	-0.4		167	166	+1.6		28.6	29.3	-0.7	
(n: 277)	(77.4-81.9)	(77.7-82.4)	(-1.0 to +0.3)		(166-168)	(164-167)	(+1.2 to +2.0)		(27.8-29.3)	(28.4-30.1)	(-1.0 to -0.4)	
Medium	79.1	79.3	-0.2		169	167	+1.6		27.8	28.5	-0.6	
(n: 282)	(76.8-81.4)	(76.9-81.7)	(-0.8 to +0.4)		(167-170)	(166-168)	(+1.0 to +2.2)		(27.1-28.6)	(27.6-29.3)	(-0.9 to -0.3)	
High	75.2	75.2	-0.04		168	167	+1.5		26.6	27.1	-0.5	
(n: 352)	(73.5-76.9)	(73.4-77.0)	(-0.6 to +0.5)		(167-169)	(166-167)	(+1.2 to +1.8)		(26.1-27.2)	(26.5-27.7)	(-0.7 to -0.3)	

**Diet^4^**				0.12				0.71				0.09
Non-veg	82.9	83.3	-0.5		168	166	+1.5		29.6	30.3	-0.7	
(n = 400)	(81.0-84.8)	(81.4-85.3)	(-1.0 to +0.1)		(167-169)	(165-167)	(+1.0 to +1.9)		(28.9-30.2)	(29.6-31.0)	(-1.0 to -0.5)	
Semi-veg	77.3	77.7	-0.4		168	167	+1.8		27.3	28.0	-0.7	
(n = 185)	(74.9-79.7)	(75.3-80.2)	(-1.2 to +0.3)		(167-170)	(165-168)	(+1.2 to +2.3)		(26.5-28.1)	(27.2-28.9)	(-1.1 to -0.4)	
Vegetarian	71.7	71.5	+0.3		168	167	+1.5		25.3	25.7	-0.4	
(n = 326)	(70.0-73.5)	(69.6-73.3)	(-0.2 to +0.8)		(167-170)	(166-168)	(+1.2 to +1.9)		(24.8-25.9)	(25.1-26.3)	(-0.6 to -0.2)	

Difference: self-reported - measured (a negative value means infra-reported; a positive value means over-reported).

The values do not sum 911 because there are 10 participants with other race. Due to limited number of participants we do not represent category.

The values do not sum 911 because there are 191 missing values for this variable. Soy consumer: ≥1 g of soy protein per day.

Semi-vegetarian: low meat consumption and pesco; Vegetarian: Vegan and Lactovegetarian.

Values are expressed as means and (95% Confidence Interval).

p values of differences between groups were obtained through T Student test or ANOVA.

Spearman correlation coefficients (95% CI) for weight, height, and BMI were 0.96 (0.95-0.96), 0.96 (0.95-0.96), and 0.94 (0.93-0.94), and intraclass correlation coefficients were 0.98 (0.97-0.98), 0.95 (0.93-0.97), and 0.97 (0.96-0.97). The sensitivity of self-reported BMI to detect obesity was 0.81 (0.76-0.85), the specificity 0.97 (0.95-0.98), the predictive positive value 0.93 (0.89-0.97), the predictive negative value 0.92 (0.89-0.94), and the Kappa index 0.81 (0.77-0.85).

After cross-classifying participants into quintiles of self-reported and measured weight, the absolute agreement between quintiles was 74% (95% CI: 71-77%). In addition, if we calculated the absolute agreement between self-report and measured values using a category including participants with values in the same quintiles or +/- 1 adjacent quintile, there was almost total agreement (99.1%, 95% CI: 98.3%-99.6%).

The absolute agreement in the cross-classification of participants according to their self-reported and measured BMI categories (normal weight, overweight and obese) was 83.4% (95% CI: 80.9%-85.8%).

**Figure 1:**
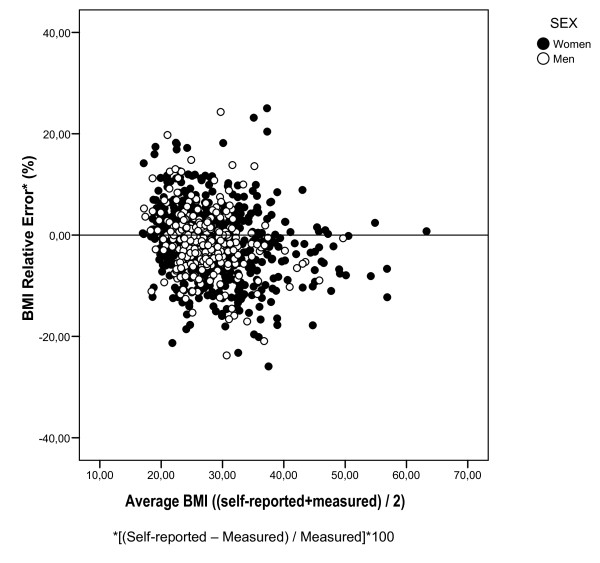
Bland-Altman plot for Body Mass Index (BMI) relative error*. *[(Self-reported - Measured)/Measured]*100.

After multivariate analyses, predictors of differences between self-reported and measured weight were age, obesity, education, soy consumption, and type of dietary pattern followed-up. Similarly, predictors of BMI differences were obesity, soy consumption, and type of dietary pattern followed-up (Table 4). The Bland-Altman plot shows a relatively random variability given the inexistence of a funnel plot (Figure 1).

**Table 4:** Multivariate Prediction of differences between self-reported and measured weight and body mass index (BMI).

	Weigh difference* (kg)			BMI difference* (kg/m^2^)	
**Variables**	**Regression coefficient (ß)** **(95% CI)**	**p value**		**Regression coefficient (ß)** **(95% CI)**	**p value**

Age					
<45 years	0 (Ref.)			0 (Ref.)	
45-<55 years	-0.82 (-1.85 to +0.21)	0.12		-0.33 (-0.77 to +0.11)	0.15
55-<65 years	+1.35 (+0.30 to +2.40)	0.01		+0.22 (-0.23 to +0.67)	0.33
65- <75 years	+1.89 (+0.75 to +3.02)	0.001		+0.25 (-0.23 to +0.74)	0.31
≥75 years	+2.29 (+0.99 to +3.58)	0.001		-0.11 (-0.66 to +0.44)	0.71
Sex (Female vs Male)	-0.14 (-0.89 to +0.60)	0.70		+0.08 (-0.24 to +0.39)	0.64
Obese (BMI≥30 kg/m^2^) (measured)	-2.73 (-3.47 to -1.98)	<0.001		-1.55 (-1.86 to -1.23)	<0.001
Ever smokers	-0.71 (-1.59 to +0.17)	0.12		-0.18 (-0.55 to +0.20)	0.36
Married	-0.44 (-1.32 to +0.44)	0.33		-0.12 (-0.50 to +0.26)	0.54
Time lag between measurements					
Quartile 1 (<1.29 years)	0 (Ref.)			0 (Ref.)	
Quartile 2 (≥1.29 - <1.58 years)	-0.07 (-1.00 to +0.86)	0.88		+0.01 (-0.38 to +0.41)	0.95
Quartile 3 (≥1.58 - <2.33 years)	-0.26 (-1.19 to +0.67)	0.59		-0.12 (-0.52 to +0.28)	0.56
Quartile 4 (≥2.33 years)	+0.59 (-0.37 to +1.55)	0.23		+0.25 (-0.16 to +0.66)	0.23
Education					
≤High School	0 (Ref.)			0 (Ref.)	
≤2-year college	-1.27 (-2.16 to -0.38)	0.05		-0.39 (-0.77 to -0.01)	0.04
≤Doctoral degree	-0.63 (-1.59 to +0.32)	0.19		-0.13 (-0.54 to +0.28)	0.53
Income per capita^1^					
Low	0 (Ref.)			0 (Ref.)	
Medium	-0.29 (-1.26 to +0.69)	0.53		-0.24 (-0.66 to +0.18)	0.26
High	-0.56 (-1.68 to +0.57)	0.33		-0.26 (-0.75 to +0.22)	0.28
Race					
White	0 (Ref.)			0 (Ref.)	
Black	+0.01 (-0.74 to +0.75)	0.99		-0.10 (-0.42 to +0.22)	0.56
Hispanic	-0.11 (-1.89 to +1.67)	0.90		-0.16 (-0.92 to +0.60)	0.68
Asian	+0.80 (-1.63 to +3.23)	0.52		+0.30 (-0.74 to +1.34)	0.29
Other	-1.27 (-4.77 to +2.23)	0.48		-0.31 (-1.81 to +1.18)	0.49
Physical Activity^2^					
Low	0 (Ref.)			0 (Ref.)	
Medium	-0.36 (-1.35 to +0.64)	0.48		-0.23 (-0.66 to +0.19)	0.29
High	-0.33 (-1.28 to +0.64)	0.51		-0.14 (-0.55 to +0.27)	0.49
Soy consumers^3^	+1.39 (+0.46 to +2.32)	0.003		+0.56 (+0.16 to +0.96)	0.006
Nuts consumption					
≤1/week	0 (Ref.)			0 (Ref.)	
2 to 4/week	-0.11 (-0.95 to +0.74)	0.80		-0.05 (-0.41 to +0.31)	0.78
≥5/week	-0.26 (-1.10 to +0.58)	0.55		-0.02 (-0.37 to +0.34)	0.94
Diet^4^					
Non-vegetarian	0 (Ref.)			0 (Ref.)	
Semi-vegetarian	-1.04 (-1.95 to -0.13)	0.03		-0.42 (-0.81 to -0.04)	0.03
Vegetarian	-0.61 (-1.44 to +0.22)	0.15		-0.25 (-0.60 to +0.10)	0.17
					

95% CI: 95% Confidence Interval.

*Difference = Self-reported - Measured.

All the variables are adjusted through a multivariate linear regression analysis for the all variables shown in the table.

1) Low: <10,000$, medium: ≥10,000 - <30,000$, High: ≥30,000$.

2) Low: < 20 minutes of non-vigorous activity; medium: ≥20 minutes of non-vigorous activity; high: ≥20 minutes of vigorous activity.

3) Soy consumers: ≥ 1 g of soy protein consumption per day.

4) Semi-vegetarian: low meat consumption and pesco; Vegetarian: Vegan and Lactovegetarian.

**Figure 2:**
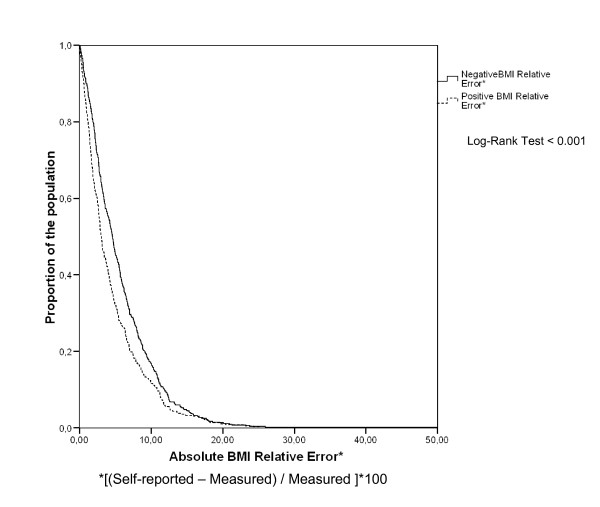
**Survival agreement Plot for Body Mass Index (BMI)**. *[(Self-reported - Measured)/Measured ]*100 Log-Rank Test < 0.001 Survival agreement Plot as proposed by Luiz et al. (J Clin Epidemiol 2003; 56:963-7). The x-axis shows the absolute difference between self-reported and measured weight (kg), and the y-axis shows the proportions of observations with differences that are at least those in the x-axis. Separate lines for negative difference (self-reported-measured; infra-reported) and continuous line for positive difference (self-reported-measured; over-reported).

Finally, we used the survival-agreement plot to depict graphically the agreement between self-reported and measured BMI (Figure 2). We noted that negative BMI relative errors (under-reported) tended to be greater than positive BMI relative errors (over-reported) (log-rank test, p < 0.001).

## Discussion

Overall our results showed a high validity of self-reported anthropometrics in a representative subsample of the AHS-2 cohort. These results are in agreement with a meta-analysis of self-reported weight that included 24 studies which concluded that self-reporting is sufficiently accurate in epidemiological studies [13]. However, in the present study we also found some independent predictors of inaccurate values after multivariate analysis.

In contrast to some previous studies [6], [14], [15] we did not find differences between sexes in evaluating anthropometric data. Nevertheless, in agreement with the majority of the studies age was a predictor of reporting less height [3], [5], [15], [16]. This is understandable, since height declines with age in later life [17] and older people may be unaware of this decrease in their stature. Additionally in our study, age was also a predictor of reporting greater weight.

Similarly to other studies [3], [5], [16], [18] obese subjects provided less accurate weight and BMI data. They tended to underestimate their weight and hence BMI, but the bias in self-reported weight was only 0.6 kg on average among the obese. In this context, and in agreement with Villanueva et al [3], normal weight participants were least likely to be incorrectly classified to another BMI category, while obese participants were most likely to be incorrectly classified since obesity is seen as socially undesirable.

Regarding education, participants with at least 2-years of college reported less weight and BMI compared to those with a high school education or lower. This may be explained by the higher pressure and desirability bias in this group of people.

Race was not an independent predictor of validity as was observed in a recent study [6]. There were no differences between the white and black population of AHS-2 in the validity of self-reported anthropometrics. Gillum, et al [4] with data from NHANES III, similar to Kuczmarski, et al. [16], also did not find differences between blacks and whites. Gillum, et al [4] found a greater bias in BMI using self-reported data from Hispanic men and women compared with white non-Hispanic men and women. This is similar to our findings even though the trends were not statistically significant probably due to the small number of Hispanics' participants.

Interestingly, we found that soy consumers over-reported their weight compared to non-soy consumers; however, the latter group was more inaccurate regarding their anthropometric data, as the variances of differences between self-report and measured data were higher. Soy consumers might be more health conscious and as a consequence they might have more accurate knowledge of their weight and height. By contrast, semi-vegetarian participants, those with low meat and fish consumption, significantly underreported their weight and BMI in comparison to non-vegetarians. Future research is necessary to determine the generalizability of these findings to other settings. It is worth noting that the magnitude of differences between self-reported and measured weight was smaller than in other populations [5], [18]. The sensitivity in assessing obesity was higher [81%] than the 74% observed in the USA [19], 57% in Spain [20] and 55-61% in Sweden [21]. Our results were similar to those in a Scottish population [22] and in a Japanese workplace population [23].

One potential limitation of the study was the existence of a relatively wide time-lag between ascertainment of self-reported data and measured data. The median time-lag between the answered questionnaire and the time when subjects were been objectively weighted and measured for height was 1.58 years. Therefore, the observed differences between self-reported and measured weight and BMI may truly be due to real changes of weight and BMI across time. In fact, when we repeated the analyses including only those participants who went to clinics within a year after answering the questionnaire the results improved (i.e. Kappa index for obesity: 0.87; 95% CI: 0.77-0.97). However, our results were adjusted for time-lag period and in any case this potential limitation should bias the results towards a worse validity. Confounding by variables not controlled for cannot be excluded. However, given the uncertainty about the existence or nature of such associations it is unclear which other variables should be controlled for as confounders.

On the other hand, subjects completed the questionnaire on self-reported weight and height before the clinics and without knowing that their weight and height would be measured in the future. In addition, the number of participants in the validation study is relatively high and it was a representative sample of the AHS-2 cohort.

## Conclusion

In conclusion, this study indicates that self-reported weight and height data from a cohort of Adventists in the USA and Canada is valid enough to be used in epidemiological association studies, although it can lead to some underestimation of obesity.

## Abbreviations

BMI: Body Mass Index; AHS-2: Adventists Health Study 2; 95% CI: 95% Confidence Interval

## Competing interests

The authors declare that they have no competing interests.

## Authors' contributions

All authors (MB-R, JS, KJ, GF) participated in the planning and conception of the research questions and the study design. GF was the principal investigator of the study and primarily conceptualized the research. MB-R analyzed the data and drafted the manuscript. JS contributed to the study design, interpretation of data, and review the paper. KJ was responsible for retrieving the data. All authors participated in interpreting the data and critically revising the manuscript for important intellectual content. All authors have approved the final version of the manuscript.

## Pre-publication history

The pre-publication history for this paper can be accessed here:

http://www.biomedcentral.com/1471-2458/11/213/prepub
